# Evaluation of the VP22 protein for enhancement of a DNA vaccine against anthrax

**DOI:** 10.1186/1479-0556-3-3

**Published:** 2005-04-20

**Authors:** Stuart D Perkins, Helen C Flick-Smith, Helen S Garmory, Angela E Essex-Lopresti, Freda K Stevenson, Robert J Phillpotts

**Affiliations:** 1 Biomedical Sciences Department, Defence Science and Technology Laboratory, Porton Down, Salisbury, Wiltshire, SP4 OJQ, UK; 2 Tenovus Laboratory, University of Southampton Hospital NHS Trust, Southampton, SO16 6YD, UK

## Abstract

**Background:**

Previously, antigens expressed from DNA vaccines have been fused to the VP22 protein from Herpes Simplex Virus type I in order to improve efficacy. However, the immune enhancing mechanism of VP22 is poorly understood and initial suggestions that VP22 can mediate intercellular spread have been questioned. Despite this, fusion of VP22 to antigens expressed from DNA vaccines has improved immune responses, particularly to non-secreted antigens.

**Methods:**

In this study, we fused the gene for the VP22 protein to the gene for Protective Antigen (PA) from *Bacillus anthracis*, the causative agent of anthrax. Protective immunity against infection with *B. anthracis *is almost entirely based on a response to PA and we have generated two constructs, where VP22 is fused to either the N- or the C-terminus of the 63 kDa protease-cleaved fragment of PA (PA_63_).

**Results:**

Following gene gun immunisation of A/J mice with these constructs, we observed no improvement in the anti-PA antibody response generated. Following an intraperitoneal challenge with 70 50% lethal doses of *B. anthracis *strain STI spores, no difference in protection was evident in groups immunised with the DNA vaccine expressing PA_63 _and the DNA vaccines expressing fusion proteins of PA_63 _with VP22.

**Conclusion:**

VP22 fusion does not improve the protection of A/J mice against live spore challenge following immunisation of DNA vaccines expressing PA_63_.

## 1.0 Background

The VP22 protein is a major component of the amorphous tegument region of the Herpes Simplex Virus type I (HSV-1). Composed of 301 amino acids, it has become known as a protein transduction domain able to mediate intercellular spread. Like other translocatory proteins such as antennapedia and the HIV Tat protein, it is highly basic, it is able to bind heparin or sialic acid and all three proteins have an almost identical predicted pI [1]. VP22 has been reported as being able to exit the cell in which it is synthesised via an uncharacterised, golgi-independent secretory pathway and subsequently enter surrounding cells by a non-endocytic mechanism. These properties may be retained after fusion to other proteins [2].

The ability to 'piggyback' proteins or peptides into cells may be particularly useful for gene therapy. Thymidine kinase and p53 have benefited from fusion with VP22 [3,4]. VP22 has been fused to proteins and delivered by a viral vector. For example, p53 delivered by an adenovirus vector [5,6], GFP delivered by a lentivirus vector [7] and Human papillomavirus E7 antigen delivered by a Sindbis replicon [8,9] have all proved more effective after VP22 fusion.

However, the ability of VP22 to mediate intercellular spread has been questioned, based on *in vitro *studies that use methanol fixation. Because methanol dissolves cellular membranes, it may produce an artefact interpreted as cell to cell spread [10]. In further studies, transport could not be detected in live cells [11] and a fusion protein of VP22 and diphtheria toxin A (a single molecule of which is lethal to a cell) could not cross the cell membrane and cause a measurable cytotoxic effect [12]. A critical analysis of the literature has led to the conclusion that the effects of VP22 can be explained by well-established biological principles whereby VP22 causes liberation from cells, possibly by cell death. Following this, the protein may bind to surrounding cells, but does not efficiently penetrate cellular membranes [1].

Irrespective of whether VP22 can mediate intercellular spread however, VP22 can enhance *in vivo *responses to a number of antigens not only in the context of gene therapy, but also when fused to antigens within a DNA vaccine. This could be particularly useful because although DNA vaccines can offer protection against a wide variety of pathogens in small animal models, their efficacy in larger animal models and primates is insufficient. In this study, we evaluate the potential of VP22 to enhance DNA vaccines against anthrax.

The spore-forming bacterium *Bacillus anthracis *causes the disease anthrax. The current UK-licensed vaccine is an alum-precipitated filtrate of a *B. anthracis *Sterne strain culture, administered by the intramuscular route, which occasionally causes some transient reactogenicity in vacinees [13]. The US-licensed vaccine is the Anthrax Vaccine Adsorbed (BioThrax-AVA) vaccine produced from the culture supernatant fraction of the V770-NP1-R strain [14].

The key component in both these vaccines is the protective antigen (PA), which along with lethal factor (LF) and edema factor (EF) forms a tripartite toxin and is one of the virulence factors of the bacteria [15]. Host cell intoxication is thought to occur after binding of the full length 83 kDa PA to the host cell membrane receptor. The 20 kDa N-terminal fragment of PA is cleaved by furin protease exposing the LF-EF binding site [16]. The 63 kDa PA fragments form a heptameric pore, the LF or EF bind and the whole toxin complex is internalised [17,18].

DNA vaccines against *B. anthracis *expressing either the 63 kDa fragment of PA [19,20] or the 83 kDa PA protein have proved successful [21]. Protection against lethal toxin challenge in Balb/c mice or a spore challenge in NZW rabbits can be achieved by either intramuscular or gene gun immunisation [19-22]. Attempts to enhance the protective efficacy of DNA vaccines against anthrax include co-administration with a DNA vaccine expressing LF, and a DNA prime / protein boost regimen [20] or the use of cationic lipids [22].

The aim of this study was to assess the potential of VP22 to enhance the immunogenicity of a DNA vaccine expressing the 63 kDa fragment of PA (PA_63_) attached to a secretion signal. The VP22 protein, which has previously been shown to improve the performance of DNA vaccines [23-26], was fused to either the N- or the C-terminus of PA_63_. We show that following gene gun administration of these vaccines, fusion with VP22 does not improve anti-PA antibody responses to the PA_63 _DNA vaccine, nor does it increase protection against anthrax lethal spore challenge.

## 2.0 Methods

### 2.1 Construction of DNA vaccines

The DNA vaccine pGPA contains the signal sequence for human plasminogen activator fused to the N-terminus of the gene for the 63 kDa fragment of PA [19] and was a kind gift from Dennis Klinman (Food and Drug Administration, USA). To include the VP22 sequence derived from amino acids 159 – 301, which possesses the full transport activity of the native protein (both the intrinsic transport ability and the ability to carry proteins of significant size [27]), the following strategy was employed. To construct the N-terminal fusion, the gene for the VP22 sequence was PCR amplified from pCR^®^T7/VP22-1 (Invitrogen) using primers VP22 F9 (5' ACTCTA*GCTAGC*ACGGCGCCAACCCGATCCAAGACA 3') and VP22 R8 (5' ATTGTCACGGTCTGGAACCGTAGGAGCAGCTGGACCTGGACCCTCGACGGGCCGTCTGGGGCGAGA 3'). Additionally, the gene for PA_63 _was PCR amplified from pGPA using primers PA F8 (5' CCTACGGTTCCAGACCGTGACAAT 3') and PA R9 (5' CGC*GGATCC*TTATCCTATCTCATAGCC 3'). The two sequences were then fused together by PCR [28] using primers VP22 F9 and PA R9.

**Figure 1:**
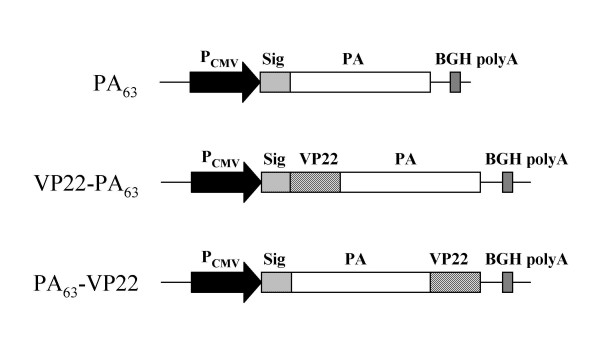
DNA vaccines constructed in this study as in section 2.1. DNA vaccine expressing PA_63 _(pGPA) is a kind gift from Dennis Klinman (Food and Drug Administration, USA). (Abbreviations: P_CMV_, CMV promoter; Sig, Signal sequence; BGH polyA, Bovine growth hormone polyadenylation signal).

To create the C-terminal fusion, the gene for the PA_63 _sequence was PCR amplified from pGPA using primers PA F11 (5' CTA*GCTAGC*CCTACGGTTCCAGACCGTGACAAT 3') and PA R10 (5' TGTCTTGGATCGGGTTGGCGCCGTAGCAGCTGGACCTGGACCTCCTATCTCATAGCC 3'). The gene for VP22 was PCR amplified from pCR^®^T7/VP22-1 (Invitrogen) using primers VP22 F10 (5' CGGCGCCAACCCGATCCAAGACA 3') and VP22 R11 (5' CGC*GGATCC*TTACTCGACGGGCCGTCTGGGGCGAGA 3'). The two sequences were then fused together using VP22 F10 and VP22 R11 by PCR fusion [28]. The PCR primers used to create the two gene fusions were designed to incorporate a linker sequence of Gly-Pro-Gly-Pro-Ala-Ala between the VP22 and PA_63 _proteins, to allow folding of the fusion protein. Using restriction sites *Nhe*I and *Bam*HI, the PA_63 _gene was exised from pGPA and the gene fusions were ligated into the vector to form pSTU-22-PA (N-terminal fusion) and pSTU-PA-22 (C-terminal fusion). These constructs were verified by sequencing and are schematically represented in figure 1.

The control DNA vaccine expressing VP22 only (pSTU22) has been previously described [26]. The plasmid DNA was prepared using Qiagen Endofree DNA purification columns (Qiagen Ltd).

### 2.2 Western blot analysis of expressed proteins

African Green Monkey Kidney COS-7 cells (European Collection of Animal Cell Cultures, Porton Down) were plated at 1–5 × 10^5 ^cells well^-1 ^into 6 well plates (Corning). Cells were transfected with 1 μg of plasmid DNA using the transfection reagent Polyfect (Qiagen) according to the manufacturer's guidelines. Transfected cell lysates were separated by 4–20% polyacrylamide gel electrophoresis (Tris-Glycine gel, Invitrogen), using XCell SureLock™ Mini-Cell apparatus (Invitrogen) according to the manufacturer's protocol. Protein from the gel was then transferred to nitrocellulose by electroblotting (Invitrogen). An ECL Western blotting kit (Amersham Biosciences) was used with antibody to PA (rabbit polyclonal sera) or VP22 (rabbit polyclonal sera) to detect expression from DNA vaccines.

### 2.3 Vaccination of Balb/c mice

Groups of 10 female A/J mice (Harlan OLAC) were immunised with 1 μg of DNA coated onto gold particles and delivered using a Helios™ gene gun (BioRad) as described previously [29]. Mice were immunised three times at two-week intervals. Blood was taken from the tail vein prior to challenge for serum antibody analysis by enzyme-linked immunosorbent assay (ELISA).

### 2.4 Measurement of anti-PA antibodies by ELISA

Microtitre plates were coated with 5 μg ml^-1 ^recombinant PA (Aldevron) in phosphate-buffered saline using 50 μl well^-1 ^and incubated overnight at 4°C. Three columns on each plate were coated with anti-IgG (Fab) (Sigma) in order to produce a standard curve for quantification of IgG concentration. After washing three times with PBS containing 0.2% Tween-20, non-specific binding was blocked with 5% (w/v) powdered skimmed milk in PBS and the plates were incubated for 2 hours at 37°C. The plates were washed three times and serum was added at a starting dilution of 1:50 in blocking buffer, and double-diluted down the plate. IgG or isotype standards (Sigma), diluted in blocking buffer, were added to wells which had been coated with anti-IgG (Fab) (Sigma), and double diluted as before. Plates were incubated for 1.5 hours at 37°C before washing and the addition of goat anti-mouse IgG (or anti-mouse IgG isotype) conjugated to horseradish peroxidase (Sigma), diluted in blocking buffer. Plates were incubated for 1 hour at 37°C, then washed 3 times before addition of the substrate ABTS (Sigma). Absorbance at 410 nm was measured after 20 minutes incubation at room temperature and analysed using Ascent software.

### 2.5 Challenge with *B. anthracis*

Three weeks after the final immunising dose, mice were challenged intraperitoneally with *B. anthracis *STI (Tox^+ ^Cap^-^) spores. Sufficient spores for the challenge were removed from stock cultures, washed in sterile distilled water, and resuspended in PBS to a concentration of 7 × 10^5 ^spores ml ^-1^. Mice were challenged with 100 μl volumes containing 7 × 10^4 ^spores per mouse (equivalent to 70 50% lethal doses [LD_50_s] [30]) and were monitored for 18 days post challenge to determine their protected status. Humane endpoints were strictly observed so that any animals displaying a collection of clinical signs that indicated a lethal infection were culled.

### 2.6 Statistical Methods

One-way ANOVA with Tukey's multiple comparison post analysis test and statistical analysis of survival using the Mantel-Haenszel Logrank test were performed using GraphPad Prism version 3.02 for Windows, GraphPad Software, San Diego, California, USA .

## 3.0 Results

### 3.1 *In vitro *expression of DNA vaccines

**Figure 2:**
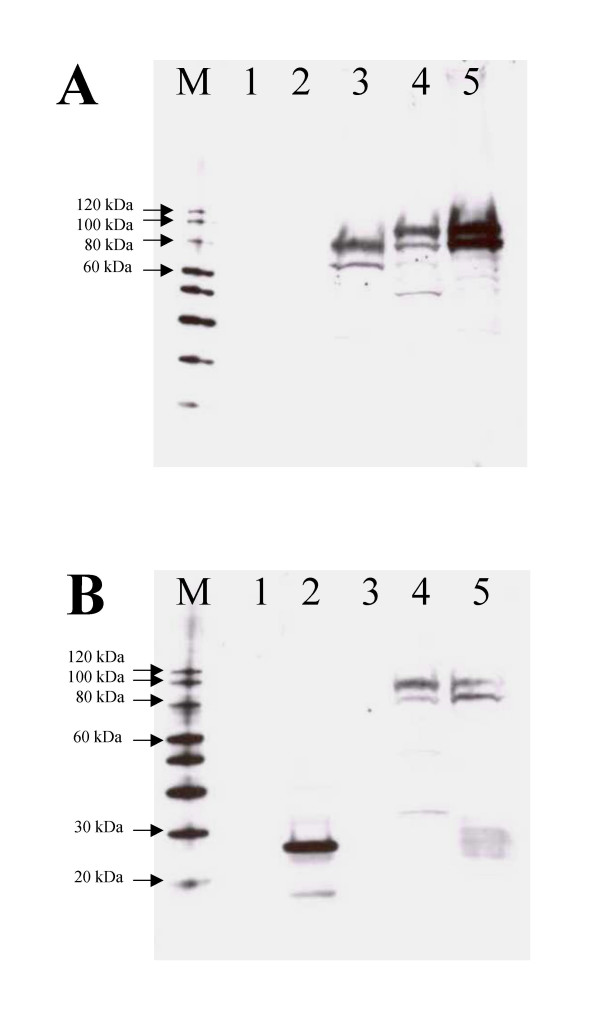
Western blot analysis of DNA vaccines. Membranes were probed with anti-PA antibody (A) or anti-VP22 antibody (B) as described in section 2.2. Cells were untransfected (1) or transfected with DNA vaccines expressing VP22 (2), PA_63 _(3), VP22-PA_63 _(4) or PA_63_-VP22 (5).

DNA vaccines encoding PA_63_, VP22-PA_63_, PA_63_-VP22 or VP22 (Figure 1) were transfected into African Green Monkey Kidney cells (COS-7). Cells were harvested and processed for Western blot analysis 48 hours post transfection. Cells transfected with the PA_63_-encoding DNA vaccine expressed a protein of approximately 68 kDa that reacted with PA-specific antibody. Fusion of VP22 to either the N-terminal or C-terminal of PA_63 _resulted in a protein of approximately 90 kDa that reacted with both PA-specific antibody and VP22-specific antibody (Figure 2). Control cells, transfected with plasmid DNA expressing VP22 only expressed a protein of approximately 22 kDa that reacted with VP22-specific antibody. Some degradation of the PA_63 _proteins was evident irrespective of whether fused to VP22 or not. However, the degraded fusion proteins were recognised by both the anti-PA and anti-VP22 antibodies suggesting that this degradation was not due to instability at the point of fusion of the two proteins.

### 3.2 Anti-PA antibody responses following gene gun immunisation

**Figure 3:**
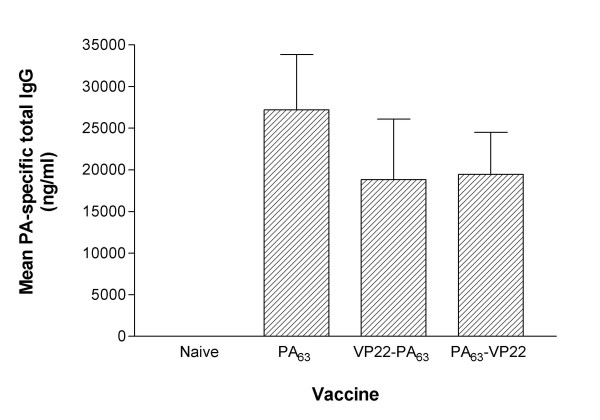
A/J mice were immunised with DNA vaccines expressing PA_63_, VP22-PA_63 _or PA_63_-VP22. Anti-PA total IgG levels in the sera at day 38 were determined by ELISA. Bars represent the mean of each group, the error bars represent 95% confidence intervals. n = 10 mice per group.

Groups of 10 female A/J mice were immunised three times by gene gun administration of 1 μg plasmid DNA at two weeks intervals. Serum samples were collected 17 days after the third immunisation (4 days before challenge). Sera from individual mice were assayed for PA-specific total IgG (Figure 3). Mice immunised with PA_63_-expressing DNA vaccine produced a mean titre of 27,216 ng/ml total PA-specific IgG, compared with 18,823 ng/ml and 19,448 ng/ml for the VP22-PA_63 _and PA_63_-VP22 -expressing DNA vaccines respectively. These antibody titres of PA-specific total IgG did not differ significantly between the three groups (p > 0.05, One-way ANOVA with Tukey's multiple comparison posthoc analysis).

### 3.3 Protection against anthrax spore challenge

**Figure 4:**
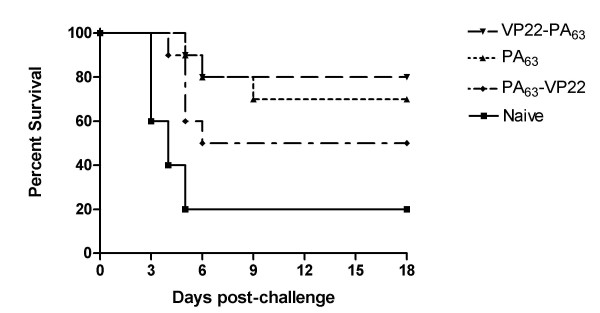
Numbers of mice surviving 18 days post challenge with 70 LD_50_s of *B. anthracis *STI spores after immunisation with DNA vaccines expressing PA_63_, VP22-PA_63 _or PA_63_-VP22. n = 10 mice per group.

Mice were challenged three weeks after the final dose with 70 50% lethal doses of *B. anthracis *strain STI by the intraperitoneal route. The DNA vaccine expressing PA_63 _conferred 70% survival to the immunised mice. In comparison, 80% and 50% of the mice survived following immunisation with the DNA vaccines expressing VP22-PA_63 _and PA_63_-VP22, respectively (Figure 4). Thus inclusion of the VP22 protein at either the N- or C-terminus of PA_63 _did not significantly alter protection of the mice. All three vaccines offered a significant level of protection compared to naïve mice. Statistical analysis of survival was performed using the Mantel-Haenszel Logrank test (GraphPad Prism).

## 4.0 Discussion

The Herpes Simplex virus type I VP22 protein has been suggested to mediate intercellular spread by exit from cells in a golgi-independent manner and entry to adjacent cells by a non-endocytic mechanism [2]. However, *in vitro *studies of this protein remain inconclusive, with reports that the apparent effects of VP22 can be attributed to an artefact produced by methodology [1,10,31,32]. Despite the controversy surrounding *in vitro *studies, most *in vivo *work shows that VP22 has a beneficial effect, particularly in the gene therapy field. Fusion of VP22 to either the pro drug-activating enzyme thymidine kinase [3] or the transcription factor p53 [5] results in an improvement in their effectiveness. Similarly, inclusion of this protein within a DNA vaccine can increase immune responses. Antigens shown to benefit from fusion to VP22 include Yellow Fluorescent Protein (YFP) [24], Enhanced Green Fluorescent Protein (EGFP) [26] and the human papillomavirus (HPV) E7 protein [23,25,33,34].

In this study, the VP22 protein has been fused to either the N- or C-termini of the Protective Antigen (PA) of *B. anthracis*. This antigen expressed from a DNA vaccine is protective against challenge with either lethal toxin (PA plus LF) in Balb/c mice [19,20] or spore challenge in New Zealand white rabbits [21]. We used an immunisation regimen and challenge dose of STI spores designed to offer significant but not full protection to anthrax challenge of A/J mice. This design would allow us to demonstrate any increased protection due to fusion of VP22 to PA within the DNA vaccine. Our results showed that the fusion of VP22 with PA_63 _at either terminus failed to significantly enhance anti-PA antibody responses compared to the PA_63 _DNA vaccine. Following challenge, all three DNA vaccines expressing either PA_63_, VP22-PA_63 _or PA_63_-VP22 offered significant protection against 70 LD_50_'s of *B. anthracis *compared to unimmunised control mice. However, the inclusion of VP22 did not significantly increase or decrease the protection afforded when compared to PA_63_-expressing DNA vaccine alone. This suggests that the fusion of VP22 to either the N- or C-terminus of PA_63 _within a DNA vaccine, does not alter either the antibody response elicited *in vivo *or the protection afforded to A/J mice following spore challenge. The longevity of the immune response or the ability of these DNA vaccines to initiate long-term protection was not evaluated in this study.

The failure of VP22 fusion to increase antibody responses to PA_63 _contrasts with other YFP, EGFP or HPV E7 antigens expressed from DNA vaccines where improvement is evident [23-26]. Furthermore, the failure to increase protection against *B. anthracis *challenge contrasts with studies involving DNA vaccines expressing HPV E7 protein where protective anti-tumour immunity was increased with VP22 fusion [33,34]. However, the DNA vaccines expressing the reporter proteins YFP and EGFP lack secretion signals and the level of enhancement afforded to the HPV E7 protein following fusion with VP22 was equivalent to that afforded by inclusion of a secretion signal [23]. The PA_63_-expressing DNA vaccine used here does contain a secretion signal.

The inclusion of a secretion signal is a commonly used strategy for DNA vaccination as liberation of the protein from the cell can increase immune responses [35-37]. The inclusion of VP22 within a DNA vaccine may enable non-secreted proteins to exit the cell thus increasing their exposure to antigen presenting cells such as dendritic cells. This is consistent with the hypothesis that VP22 does not mediate intercellular spread as first described, but rather is liberated from cells possibly by cell death [1]. Apart from liberation of the expressed protein from the cell, VP22 may enhance DNA vaccines in other ways. For example, the fusion of immunostimulatory sequences to antigens expressed from DNA vaccines has been shown to provide cognate T cell help [38]. In this study, a DNA fusion vaccine against B cell tumours uses the non-toxic C fragment of tetanus toxin. So it is possible that fusion of VP22 to antigens encoded by DNA vaccines may improve immunogenicity by provision of cognate T cell help.

## 5.0 Conclusion

This study investigates the inclusion of the VP22 protein in a DNA vaccine expressing PA_63 _of *B. anthracis*. The VP22 protein has been shown previously to enhance the performance of DNA vaccines expressing non-secreted proteins. In this case, the PA_63_-expressing DNA vaccine contains the human plasminogen activator signal sequence [19]. Inclusion of VP22 within this DNA vaccine construct did not enhance anti-PA antibody responses or offer an increase in the level of protection afforded to A/J mice following anthrax spore challenge. This suggests that although VP22 can improve responses to DNA vaccines encoding non-secreted proteins, it does not improve responses to a PA_63_-expressing DNA vaccine encoding a secretion signal.

## Competing interests

The author(s) declare that they have no competing interests.

## Authors' contributions

SDP, HCF-S, HSG, AEE-L carried out the studies. FKS, RJP participated in the design of the study. All authors read and approved the final manuscript.
